# Alginate vs. Hyaluronic Acid as Carriers for Nucleus Pulposus Cells: A Study on Regenerative Outcomes in Disc Degeneration

**DOI:** 10.3390/cells13231984

**Published:** 2024-11-30

**Authors:** Shota Ogasawara, Jordy Schol, Daisuke Sakai, Takayuki Warita, Takano Susumu, Yoshihiko Nakamura, Kosuke Sako, Shota Tamagawa, Erika Matsushita, Hazuki Soma, Masato Sato, Masahiko Watanabe

**Affiliations:** 1 Department of Orthopedic Surgery, Tokai University School of Medicine, 143 Shimokasuya, Isehara 259-1193, Japanschol.jordy@gmail.com (J.S.); sato-m@is.icc.u-tokai.ac.jp (M.S.); masahiko@is.icc.u-tokai.ac.jp (M.W.); 2 Center for Musculoskeletal Innovative Research and Advancement (C-MiRA), Tokai University Graduate School, 143 Shimokasuya, Isehara 259-1193, Japan; 3 TUNZ Pharma Corporation, Osaka 541-0046, Japan; takayuki.warita@tunzpharma.co.jp (T.W.); kahiko@is.icc.u-tokai.ac.jp (Y.N.); soma.hazuki@tunzpharma.co.jp (H.S.); 4 Department of Radiology, Tokai University Hospital, 143 Shimokasuya, Isehara 259-1193, Japan; taka-0336@tokai-u.jp; 5 Department of Medicine for Orthopaedics and Motor Organ, Juntendo University Graduate School of Medicine, Tokyo 113-8421, Japan; s-tamagawa@juntendo.ac.jp

**Keywords:** intervertebral disc, disc degeneration, cell therapy, biomaterials, hyaluronic acid, alginate, back pain, animal model

## Abstract

Intervertebral disc degeneration is a leading cause of chronic low back pain, affecting millions globally. Regenerative medicine, particularly cell-based therapies, presents a promising therapeutic strategy. This study evaluates the comparative efficacy of two biomaterials—hyaluronic acid (HA) and alginate—as carriers for nucleus pulposus (NP) cell transplantation in a beagle model of induced disc degeneration. NP cells were isolated, cultured, and injected with either HA or alginate into degenerated discs, with saline and non-cell-loaded carriers used as controls. Disc height index, T2-weighted MRI, and histological analyses were conducted over a 12-week follow-up period to assess reparative outcomes. Imaging revealed that both carrier and cell-loaded treatments improved outcomes compared to degenerative controls, with cell-loaded carriers consistently outperforming carrier-only treated discs. Histological assessments supported these findings, showing trends toward extracellular matrix restoration in both treatment groups. While both biomaterials demonstrated reparative potential, HA showed greater consistency in supporting NP cells in promoting disc regeneration. These results underscore HA’s potential as a superior carrier for NP cell-based therapies in addressing disc degeneration.

## 1. Introduction

Cellular therapies represent a promising frontier in the treatment of complex and traditionally intractable diseases [1,2]. By harnessing the regenerative potential of living cells, these therapies offer a novel mechanism for restoring parts of organs through remodeling damaged extracellular matrix (ECM), attracting desired cells, or modulating the local microenvironment to enhance tissue repair and functionality. Previously unmendable organs, such as nervous- [3] and cartilaginous tissues [4], are now the focus of renewed therapeutic interest as regenerative strategies emerge. One such prime target for cell therapy is the intervertebral discs (IVDs) of the spine [5,6]. 

The IVDs are fibrocartilaginous tissues that provide biomechanical integrity, stability, and flexibility to the spine, playing a crucial role in human posture and mobility. The discs are generally divided into three distinct tissue structures: the annulus fibrosus (AF), consisting of aligned collagen-fiber layers laterally enclosing the discs; the nucleus pulposus (NP), forming the center of the disc, which is highly enriched in water attractant proteoglycans; and the endplates, which form the borders of the disc with the vertebral bodies [7]. The IVDs are largely avascular [8,9], such that disc cells depend on nutrient, gas, and waste exchange through imbibition primarily across the endplates [10,11]. Given the lack of vascularization, the overall disc environment [8,12], especially the NP, is relatively nutrient-poor and characterized by harsh acidity, hypoxia, and hyper-osmolarity [10,13,14]. Coupled with the limited capacity to recruit cells from circulation and severe biomechanical stresses [15,16], the IVD creates a challenging environment for cell survival, necessitating specialized cellular adaptations [17,18]. Consequently, this suboptimal environment, coupled with aging, lifestyle factors, and wear and tear, is speculated to have detrimental effects on resident NP cells over time, leading to a decrease in cell numbers or a shift toward more catabolic phenotypes [19,20,21,22,23,24]. As this deterioration progresses, it alters the disc’s overall ECM quality and organization, creating an inflammatory milieu that further compromises the disc’s biomechanical integrity and impedes nutrient flow [10,11,25]. This creates a vicious cycle, placing additional strain on the already stressed cells [26]. This progressive cascade of catabolic events, known as disc degeneration, may ultimately lead to structural failure and impaired spinal function [27]. Specifically, the extrusion of NP tissue, the inflammation of nearby neural tissue by disc-derived inflammatory factors, or nerve ingrowth into the IVD are some of the processes believed to turn the otherwise uninnervated disc into a significant source of pain [27,28,29]. Thereby, forming one of the main sources of chronic low back pain (LBP), the primary cause of disability worldwide [22,30,31,32,33]. 

Contemporary treatments for LBP do not address the underlying decline and deterioration of disc cells and instead focus primarily on palliative care or the removal of the disc tissue, such as through spinal fusion [27,34,35]. Cell therapy presents a novel approach by directly addressing one of the root causes of the pathology and, consequentially, its symptoms. Specifically, cell products are anticipated to repopulate the disc, contribute to tissue remodeling, and promote a more anabolic environment, thereby alleviating the source of discogenic pain [36,37,38]. Several clinical trials have highlighted the potential of cell injections to reduce pain and disability, and even signs of disc regeneration on imaging modalities [5]. However, these trials typically involve first-generation cell products and are still in the early stages of development [5,39,40,41]. 

Notably, as part of disc degeneration, the overall disc environment is suboptimal for cells to thrive, which may also present a challenge for transplanted cells to survive and function effectively [25,42,43]. Specifically, the ECM environment might provide unfavorable cues to the transplanted cells, potentially inducing a catabolic phenotype [15,24,43,44,45]. Similarly, inflammatory factors and compromised nutrition within the disc may limit the potential of transplanted cells [10,42,46]. To address these issues, it has been suggested that reintroducing cells using biomaterials that can support and guide the transplanted cells in the initial post-transplantation phase may enhance the therapeutic and regenerative potential of the cell products [47,48,49]. Additionally, such biomaterials could help anchor the cells in the disc, reducing cell leakage and minimizing unintended effects elsewhere [50,51]. Despite these anticipated benefits, a systematic review by Schol et al. [5] found that few clinical trials utilize biomaterial carriers for their cell transplantation products. Furthermore, direct comparisons between different biomaterials and their impact on cell transplant potency are lacking. Therefore, there is a scientific need to evaluate various biomaterials on their ability to promote desirable outcomes for cell-mediated disc repair.

In this study, we selected hyaluronic acid (HA) and alginate as biomaterial carriers for NP cell transplantation based on their unique properties and potential alignment with the challenges of disc regeneration. HA, a natural component of the IVD-ECM, offers anti-inflammatory, anti-oxidative, and ECM-modulating effects while supporting hydration and structural integrity within the disc [52,53,54,55,56]. These features, along with promising pr-clinical and clinical outcomes demonstrating HA’s ability to enhance cell viability and promote disc regeneration, make it a leading candidate for cell-based therapies [52,57,58,59]. Alginate, derived from brown seaweed, is widely recognized for its biocompatibility, ability to mimic the native ECM, and capacity to form a supportive three-dimensional network that maintains tissue hydration and provides mechanical stability [60,61,62]. While limited clinical data exist for alginate, preclinical studies have highlighted its potential to deliver therapeutic agents effectively and mitigate disc degeneration and inflammation [61,63,64]. Thus, we aim to review two of the most commonly studied biomaterials within IVD repair and assess their ability to support our NP cell-based therapeutic as a treatment to reduce or reverse the progression of disc degeneration [65]. We applied both biomaterial carriers in a clinically relevant beagle model of induced disc degeneration [66,67] and assessed the cell and biomaterial products to mitigate the detrimental effects of the induced disc degeneration.

## 2. Materials and Methods

### 2.1. Ethical Considerations and Approval

The study described below adheres to all national and institutional guidelines for the usage of experimental animals. The Tokai University School of Medicine Committee for Safe Animal Experimentations reviewed and approved the study design prior to its initiation (Application identifier 201098 and 212027). In a similar manner, for the collection of human NP tissue, we obtained approval from the Institutional Review Board of Tokai University School of Medicine (Application identifier 17R173) to use patients’ surgical waste tissue for scientific purposes. The patient has provided their voluntary and informed consent.

### 2.2. NP Cell Isolation, Culture and Cryopreservation

The methods employed for the creation of our cellular product followed the work of Sako et al. [59,68] and are described following the Orthopaedic Research Society (ORS) Spine section recommendations [69]. In short, NP tissue was washed, fragmented, and cultured as tissue fragments in a specialized media blend (28% F10, 41% DMEM, 30% fetal bovine serum, with 1% penicillin/streptomycin) from TUNZ Pharma (Osaka, Japan). The tissue was incubated for 14 days at 37 °C in a physioxia environment (5% CO_2_, 5% O_2_).

Next, the whole tissue cultures were digested sequentially using 0.25% Trypsin-EDTA (Themo-Fisher, Waltham, MA, USA) and 0.25 mg/mL Collagenase P (Roche, Switzerland) for 30 min and 240 min, respectively, at 37 °C. The cell yield was filtered through an 80 µm cell strainer and cultured for an additional week in TUNZ Pharma-media with 10 ng mL^−1^ FGF-2 (PeproTech, Cranbury, NJ, USA) at physioxia condition. The resulting product was collected using Trypsin/EDTA detachment and the majority of cell yield was cryopreserved in Cryostar-10 (CS-10; [STEMCELL Technologies, Vancouver, BC, Canada]) as 500,000 cells 50 µL^−1^ [70]. Cryopreserved samples were kept frozen until the time of transplantation [66]. 

### 2.3. Flow-Cytometry Assessment

The remnant cells were subjected to flow cytometry (FCM) assessment to determine the proportion of NP cells positive for NP progenitor cell and mature NP cell markers [71,72]. In short, it entailed the incubation of single-cell suspensions with either mouse anti-human Tie2 conjugated with APC (monoclonal IgG1, FAB3131A, R&D Systems, Minneapolis, MN, USA), mouse anti-human GD2 conjugated PE (Monoclonal IgG2a, 562100, BD BioSciences, San Diego, CA, USA), or mouse anti-human CD24 conjugated with FITC (Monoclonal IgG2a, 555427, BD BioSciences). Analysis was performed through FACS Calibur FCM (BD BioSciences). Only living cells were considered for analysis. Dead cells were excluded through propidium iodide staining and associated gating. 

### 2.4. Canine Disc Degeneration Model

A total of 10 female chondrodystrophic beagle dogs [67] were obtained at approximately 1 year of age from Kitayama Lab Co., Ltd., Nagano, Japan, and were acclimated to their new kennel in our research department for at least 2 weeks. The dogs had ad libitum access to water and were provided with daily feeding and washing. Body weight was monitored throughout the experimental study.

Prior to inducing disc degeneration, all dogs underwent radiographic imaging to establish baseline values. On the day of the degeneration surgery, the dogs were fasted for 12 h and then sedated with an intramuscular injection of 0.02 mg/kg Medetomidine (Kyoritsu Seiyaku Corp., Tokyo, Japan), 0.4 mg/kg Midazolam (Astellas Pharma Inc., Tokyo, Japan), and 0.4 mg/kg Butorphanol tartrate (Meiji Seika Pharma Inc., Minami-Soma, Japan). Once sedation was confirmed, the dogs were maintained under deep sedation with 2.5% Isoflurane (Pfizer, New York City, NY, USA) vapor. They were kept on a heated pad, and their vitals were continuously monitored throughout the procedure. The surgical approach was made from the left lateral side.

Disc degeneration was induced following Hiraishi et al. [66] at lumbar levels L3/L4, L4/L5, and L5/L6. Each experimental disc was surgically exposed. Next, using fluoroscopy (DHF-105CX [Hitachi, Tokyo, Japan]), an 18-gauge needle (Nipro, Osaka, Japan) was inserted and confirmed to be centrally placed in both sagittal and dorsal planes. The needle was connected to a 10 mL syringe, and by repeated suction (three times), NP tissue was extracted from the discs. The weight of the tissue collected per disc was measured immediately. Each dog received a single 0.05 mg/kg buprenorphine hydrochloride (Otsuka Pharmaceutical, Tokyo, Japan) subcutaneous injection as a prophylactic analgesic measure. Following NP aspiration procedures, all three exposed discs were sutured shut, and the dogs were moved to a heated cage, where they recovered until fully awake. They were then returned to their kennel, ending their fasting period. The induced degeneration was allowed to progress for approximately 2 weeks before transplantation was performed. 

### 2.5. Transplantation Procedure

Two weeks following the induction of disc degeneration, the canines and discs were randomly assigned to the experimental groups, with adjustments made to account for the weight of the aspirated NP tissue extracted. Discs were assigned to receive either a saline (Sham), HA, HA with cells, alginate, or alginate with cells injection. After confirmation of disc degeneration through imaging modalities, following the previous description, the canines were kept sedated and in a surgical position. A 22-gauge needle was inserted subcutaneously and confirmed to be centrally placed in the dorsal and sagittal planes. 

During the surgery, a surgical assistant prepared the transplantation products. The cryopreserved off-the-shelf (OTS) cell products were taken from the liquid nitrogen storage and kept on ice for a period of 1–2 h at maximum. For the saline transplantation, 100 µL of phosphate-buffered saline was prepared. For the HA group, the commercial 1% HA solution (ARTZ Dispo^®^ [Seikagaku Corp., Tokyo, Japan]) was mixed with CS-10 in a 1:1 ratio before injection. For the HA with cells group, the 1% HA solution was mixed with the OTS cell product in a 1:1 ratio. For the alginate group, 1% alginate (Kimica Corp., Tokyo, Japan) was mixed with CS-10, loaded with or without the OTS cell product, in a 1:1 ratio. CS10 as the suspension agent involves a serum-free, animal-free, cGMP-manufactured product containing 10% dimethyl-sulfoxide (DMSO) [70]. The onsite prepared transplantation products were handed to the performing surgeon, who was blinded to the syringe contents, disc assignments, and dog identities. The surgeon was instructed to slowly inject 100 µL of the product into the disc and maintain the syringe in place for at least 30 s before retracting the needle. Following the procedures at the three lumbar levels, similar to the degeneration process, the dogs were allowed to recover post-surgery. Each dog received a single 0.05 mg/kg buprenorphine subcutaneous injection as a prophylactic measure. Additionally, each dog was administered 0.15 mg/kg tacrolimus hydrate (Astellas, Tokyo, Japan) for 2 weeks, approximately 5 days per week, to prevent an immune response to the xenogeneic cell transplantation product [16,66]. The canines were followed for a period of 3 months prior to euthanasia to allow the biomaterial and cell products to effectuate the anabolic and potentially regenerative effects.

### 2.6. Radiographic and Magnetic Resonance Imaging

Lateral radiographic images were obtained from each canine prior to degeneration, 2–5 days before transplantation, and about 4, 8, and 12 weeks following transplantation surgery. Similarly, magnetic resonance imaging (MRI) was obtained for each canine 1 week prior to transplantation and 4, 8, and 12 weeks following transplantation. In either case, the canines were sedated as previously described, and for MRI, the canines were kept sedated using continuous isoflurane vapor. Following imaging procedures, the canines were recovered from their sedation by a single intramuscular injection of 0.1 mL 0.05 mg/kg Atipamezole (Kyoritsu Seiyaku Corp., Tokyo, Japan) as a sedative antagonist. Radiographic images were obtained using a DHF-105CX fluoroscopic imaging intensifier (Hitachi, Tokyo, Japan) with settings of 80 kV and 2 mA from a left lateral approach. 

Disc height index (DHI) was calculated based on the method described by Hiraishi et al. [66], with the baseline DHI (prior to degeneration induction) set to 100%. In brief, the methodology involved blinded measurements of the length of both the cranial and caudal vertebrae of each neighboring experimental IVD at approximately one-quarter, one-half, and three-quarters of the vertebrae’s width, following the contour of the spine. Similarly, the length of the IVD was measured at three spots. The three measurements of the IVD were summed, multiplied by two, and then divided by the summation of the six vertebral height measurements, yielding the DHI. This value was then calculated as a percentage of the baseline DHI.

MRI scans were acquired in the sagittal and axial planes using T2-weighted (T2W) imaging and T2 mapping with a 1.5T MRI system (Ingenia Ambition and Ingenia; Philips, Eindhoven, The Netherlands). The MRI settings included a TE of 150 ms, TR of 4000 ms, and FS of 1.5. T2W images were uploaded into HOROS v3.3.6, which was used to measure the general intensity indicative of the T2 relaxation time of the NP area. Using an equal area for each disc, the central area of the disc with peak intensity was measured and normalized to the intensity of the cerebrospinal fluid. Additionally, the discs were scored using the modified Pfirrmann classification scheme [73,74]. All evaluations were conducted in a blinded manner. 

### 2.7. Hematology Screening

Blood samples were collected from the canine hind paws both pre-transplantation and at 4 weeks post-transplantation to screen for any hematological abnormalities in response to the carrier or cell products. The screening was conducted by our in-house clinical hematology department.

### 2.8. Tissue Explantation and Processing

Following 12 weeks, each dog subject was sedated and humanely euthanized in accordance with institutional guidelines. An intracardiac injection of over 300 mL of 50 mg/mL pentobarbital sodium salt (P0776, Tokyo Chemical Industry Co., Tokyo, Japan) was administered. Once vital signs had ceased, the entire lumbar segment was surgically excised. Each of the experimental discs, (L3/4-L5/6) along with the healthy reference control discs (L2/3), were carefully isolated as a single motion segment by making axial cuts through the corresponding vertebrae [66,75]. 

Each tissue explant was fixed for 7 days at 4 °C in 10% formalin solution (WAKO, Japan). Next, the discs were incubated for 2 weeks in Decalcifying solution A (WAKO), which involves acid-based decalcification. The acid was neutralized by 5% sodium sulfate solution for 24 h, after which the samples were washed in a sequential 24 h of 70%, 80%, and 96% ethanol solution. Finally, the discs were cut open along their median plane and the macroscopic organization was photographed using an iPhone 12 Pro Max camera (Apple, Cupertino, CA, USA). Photographs were blinded and scored using the Thompson grading scheme [76]. 

The exposed disc explants were thereafter agitated and paraffinized. Sections were created at 8 µm thickness and prepared for histology. Each section was stained through standard Meyer’s hematoxylin solution followed by eosin for Hematoxylin and Eosin (H&E) staining. Additionally, sequential staining was performed with a 1 g/L Safranin-O solution and a 0.08% Fast-Green solution for Safranin-O/Fast-Green staining. Multiple images were captured using a KEYENCE BZ-9000 microscope (KEYENCE, Osaka, Japan) and digitally stitched with the corresponding software. The images were then blinded and evaluated according to the ORS Spine scoring criteria recommended for large animals [75]. 

## 3. Results

### 3.1. Cell Products

In this study, human NP cells were obtained from a 16-year-old male patient undergoing microdiscectomy. The isolated cells were cultured in a pre-defined culture condition [68] and subsequently harvested and divided into two fractions. One portion was cryopreserved as an OTS transplantation product. The remaining cell fraction was analyzed using FCM, which revealed the final product to have 20.1% Tie2-positivity, 16.6% GD-2 positivity, and 14.2% CD-24 positivity [71].

### 3.2. Degeneration Induction

**Figure 1:**
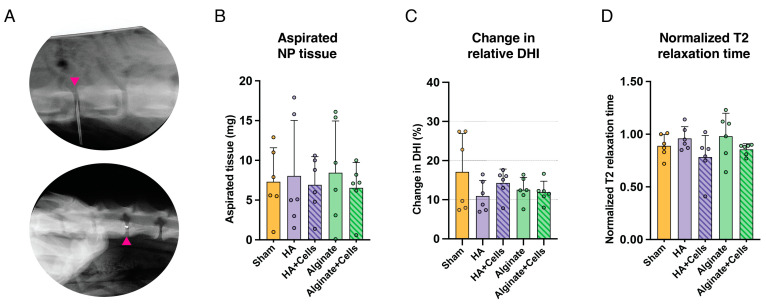
Overview of results pertaining to induced disc degeneration. (**A**) Radiographic images showing central needle placement in the disc (top; dorsal view, bottom; sagittal view), following the method described by Hiraishi et al. [66]. Magenta arrowheads point to the needle. (**B**) Assessment of NP tissue weight extracted from discs showed no significant differences in the amount of tissue extracted between conditions. (**C**) Overview of the relative disc height index (DHI) and (**D**) MRI-observed hydration loss 2 weeks after induced degeneration (i.e., at the time of transplantation) indicated no significant differences in normalized sagittal T2-relaxation times per group. For all conditions, a sample size of six was involved. Bars represent mean values, dots indicate individual outcomes per disc, and error bars denote standard deviations.

Disc degeneration was induced in 10 female beagle discs at lumbar levels L3-4, L4-L5, and L5-L6 (thus involving 30 discs in total) by aspirating NP tissue 2 weeks prior to the transplantation procedure. (Figure 1A) As part of the induction procedure, an average of 7.5 mg (±4.8 mg) wet weight NP tissue was extracted. (Figure 1B) After 2 weeks of follow-up observation, an average of 13.4% (±5.4%) relative DHI loss was observed compared to baseline, with no significant differences between conditions. (Figure 1C) Sagittal T2W images showed no significant differences between conditions in normalized T2 relaxation times at the time of transplantation. (Figure 1D) These findings confirmed successful disc degeneration at equal rates between different conditions, validating the disease model and confirming approximately equal levels of disc degeneration induced at the time of transplantation.

### 3.3. Cell Transplantation

#### 3.3.1. Surgical Procedure

After confirmation of successful disc degeneration, each experimental disc was randomly assigned to one of five treatment products, i.e., saline (Sham), HA, HA loaded with OTS NP cells, alginate, or alginate loaded with OTS NP cells. In all cases, 100 µL was injected per disc, and cell-loaded gels contained approximately 5 × 10^5^ cells. The transplantation per dog (involving three discs per animal) took on average 9 min (ranging from 7 to 12 min). All procedures were successfully performed without any complications occurring during or after the procedure. No samples were excluded from the analysis. 

#### 3.3.2. General Observations

Following transplantation, all canines were monitored for a period of 12 weeks. None of the dogs exhibited any noticeable behavioral changes, and body weight tracking did not reveal any clear issues (Supplementary Figure S1). Blood screenings conducted before and 4 weeks after transplantation showed no significant or concerning alterations (Supplementary Table S1). These observations suggest that the biomaterial and OTS cellular products were generally well-accepted.

#### 3.3.3. Disc Height Index Changes

**Figure 2:**
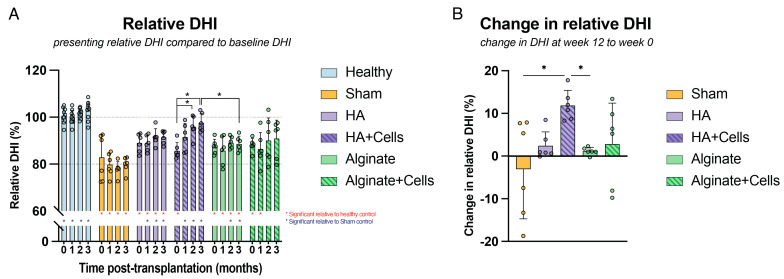
Overview of disc height index (DHI) outcomes. (**A**) Relative DHI measurements were tracked over time for each condition, with values calculated relative to baseline DHI (i.e., before disc degeneration induction). (**B**) The measured change in relative DHI at the final time point (12 weeks) was compared to the time of transplantation. * *p* < 0.05; * (red) indicates significant differences to the healthy controls at the same time point, * (blue) indicates significant differences to the sham controls at the same time point, and * (black) indicates significant differences between the indicated comparisons. Statistical analysis was performed using (**A**) 2-way ANOVA with Geisser-Greenhouse correction and (**B**) the Kruskal-Wallis test. For all conditions except the healthy controls (n = 10), a sample size of six was involved. Bars represent mean values, dots indicate individual outcomes per disc, and error bars denote standard deviations.

Throughout the 12-week observation period, monthly radiographic DHI assessments were performed. Results are presented in Figure 2A. Relative DHI measurements indicated that healthy discs maintained their DHI throughout the study. Following the initial 2 weeks, there was a slight deterioration in DHI for the sham control, whereas alginate-treated discs maintained their DHI. Discs treated with HA showed a slight trend of improvement over time. In these cases, relative DHI was significantly lower than that of the healthy controls at each time point. Conversely, the relative DHI for HA and alginate-treated discs was significantly higher than the sham control at months 2 and 3, respectively. Alginate gels loaded with OTS NP cells showed mixed results: while some treated discs exhibited deterioration in DHI, the majority of discs demonstrated improvement over time. Likely due to this variability, no significant differences were observed compared to the sham control. Discs treated with cell-loaded HA showed the most notable changes, with a significantly higher relative DHI compared to the sham control starting from month 1 and showing significant increases in DHI from month 0. Finally, when comparing the change in relative DHI at the 12th week, HA loaded with cells demonstrated a significant improvement compared to both the sham control and the alginate-treated discs (Figure 2B).

#### 3.3.4. Magnetic Resonance Imaging

**Figure 3:**
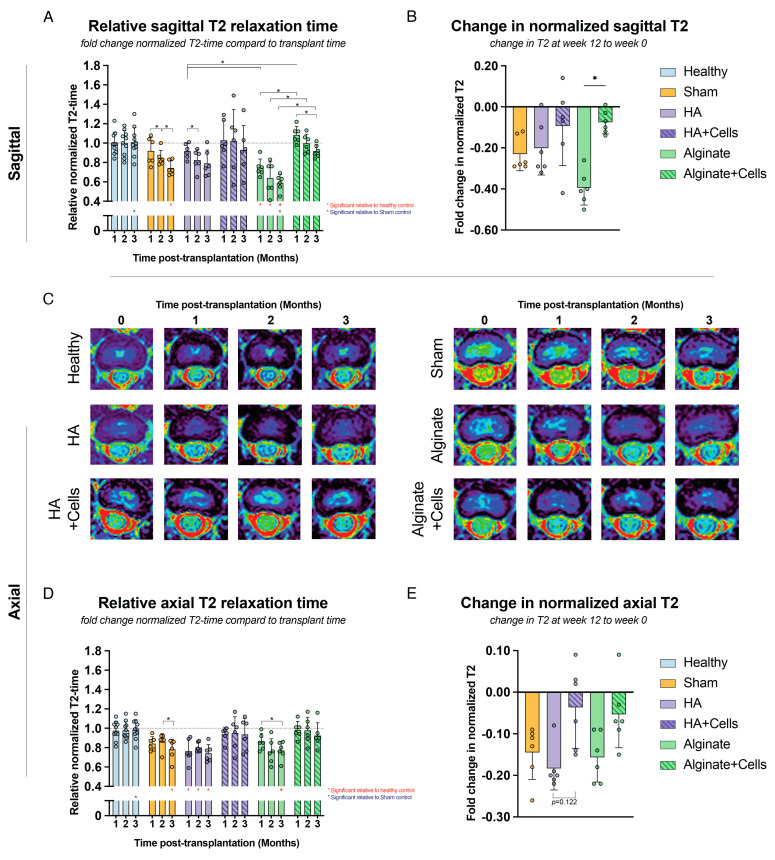
Overview of MRI results. (**A**,**D**) Relative and normalized T2-relaxtion time measurements of (**A**) sagittal and (**D**) axial images calculated as fold-change to measurements taken just prior to transplantation (thus already degenerated). (**B**,**E**) The measured change in relative normalized T2 relaxation time of (**B**) sagittal and (**E**) Axial images at the final time point (12 weeks) compared to the time of transplantation. (**C**) Representative images of axial T2 maps images obtained at 0, 1, 2, and 3 months post-transplantation. * *p* < 0.05; * (red) indicates significant differences to the healthy controls at the same time point, * (blue) indicates significant differences to the sham controls at the same time point, and * (black) indicates significant differences between the indicated comparisons. Statistical analysis was performed using (**A**,**D**) 2-way ANOVA with Geisser-Greenhouse correction and (**B**,**E**) the Kruskal-Wallis test. For all conditions except the healthy controls (n = 10), a sample size of six was involved. Bars represent mean values, dots indicate individual outcomes per disc, and error bars denote standard deviations.

In addition to radiographic imaging, MRI scans were performed just before transplantation and at approximately 4, 8, and 12 weeks post-transplantation (Figure 3). Across all conditions, a general decline in T2 relaxation time values was observed over time, indicating progressive disc deterioration. However, the transplantation products had distinct effects on the overall hydration levels of the discs. Observations on sagittal images (Figure 3A,B) showed that while the healthy control group maintained their baseline hydration values, the sham group experienced a significant reduction in T2 relaxation times, with a fold change of 0.743 (±0.074) by the final follow-up. The alginate-treated group showed an even greater decline, with a fold change of 0.592 (±0.084) at week 12. Interestingly, discs treated with alginate combined with cells significantly outperformed the carrier-only group, showing a fold change of 0.918 (±0.067) at week 12. A similar trend was observed in the HA and HA with cells treatment groups, although the results were less consistent. The HA-only group had a final fold change of 0.790 (±0.252), while the HA with cells group achieved 0.932 (±0.252). Notably, in the HA with cells group, there was a high degree of variability, with some discs showing markedly better or worse responses, leading to a broader range of outcomes. Modified Pfirrmann grades [73,74] were similarly classified, but revealed no evident differences between controls or experimental conditions (Supplementary Figure S2).

For the axial images, similar trends were observed to those in sagittal images; however, the overall rates of decline were less severe. (Figure 3C–E) Nonetheless, the general observations and subsequent measurements highlighted a clear trend of enhanced outcomes for the cell-loaded groups compared to their carrier-only cohorts. Moreover, only the cell-loaded alginate and the cell-loaded HA-treated discs were able to show a clear trend of enhanced outcomes compared to the sham control. 

#### 3.3.5. Macroscopic Images

**Figure 4:**
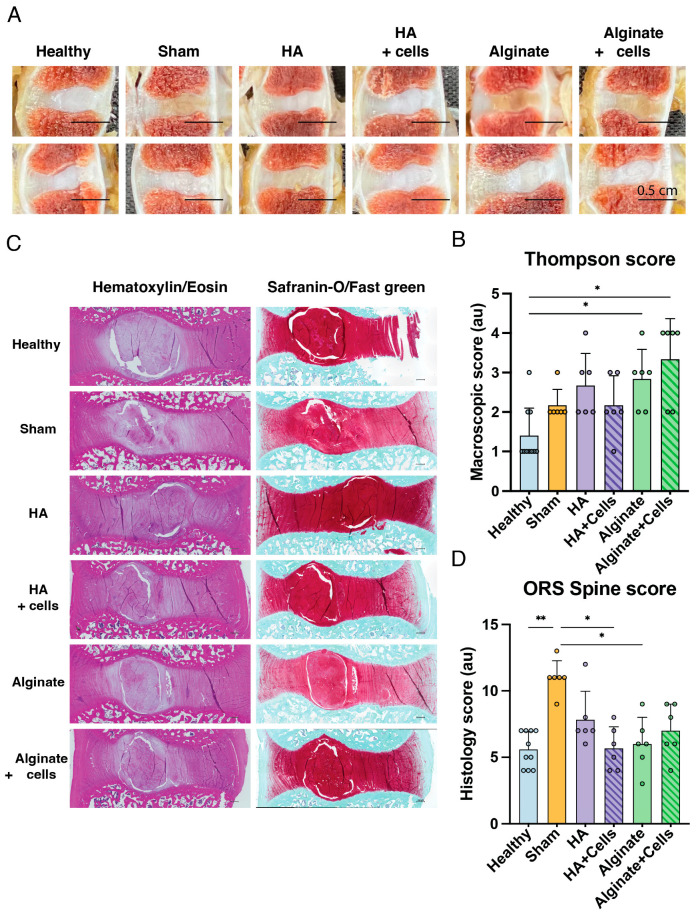
Overview of macroscopic examination. (**A**) Overview of macroscopic pictures of discs explanted of two representative samples for each condition. Scale bar represents 0.5 cm. (**B**) Thompson scores [76] given to all explanted discs 12 weeks following transplantation. (**C**) Overview of histological images of explanted discs stained with hematoxylin/eosin (**left**) and Safranin-O/Fast green (**right**). Scale bars represent 500 µm. (**D**) ORS Spine histological scores [75] given to explanted discs 12 weeks following transplantation. For all conditions except the healthy controls (n = 10), a sample size of six was used. Bars represent mean values, dots indicate individual outcomes per disc, and error bars denote standard deviation. Statistical assessment made by Kruskal-Wallis test. * *p* < 0.05, ** *p* < 0.01.

Twelve weeks following transplantations, the canine subjects were euthanized, and treated discs, as well as the L2-L3 healthy control, were excised for macroscopic and histological assessment. Explanted discs were scored in a blinded manner using the Thompson grading scheme [76]. (Figure 4A,B) This classification revealed very limited changes in overall degeneration grades. Overall, the Sham, HA, and HA loaded with cells treated discs scored similarly. Discs treated with alginate or alginate with cells showed a slight but significant increase in degenerative scores compared to healthy controls. 

#### 3.3.6. Histological Assessment

Histological observations suggest mild to moderate levels of degeneration induced overall (Figure 4C), as indicated by an average ORS Spine histological grade [75] of 11.0 (±1.3, *p* = 0.003 compared to healthy controls) for the sham conditions. (Figure 4D) All treatment conditions showed a trend of reduction in degenerative outcomes. HA-treated discs scored 7.8 (±2.1, *p* > 0.999), and alginate 6.0 (±2.0, *p* = 0.033). Similarly, cell-loaded HA resulted in 5.7 (±1.6, *p* = 0.013), while alginate with cells scored 7.0 (±2.0, *p* = 0.368). No significant differences were found between the different treatments. 

## 4. Discussion

In this study, our primary aim was to assess the potential of different biomaterials to enhance the potency of our OTS NP cell products in limiting or reversing disc degeneration in a canine model. We demonstrated that, in all cases, both the biomaterials and the biomaterials loaded with cells promoted trends of improved outcomes in imaging and histological observations compared to our negative control, which involved a saline injection. These results confirm that regenerative medicine using biomaterials or cell products has the potential to limit induced disc degeneration in preclinical models [13,77]. Notably, however, there were no significant differences between our treatment conditions; both alginate and HA products resulted in similar outcomes throughout our study. Although the differences were not statistically significant, we did observe that discs treated with cells encapsulated in HA consistently showed the most beneficial outcomes with less variability. Occasionally, these outcomes were significantly better than those observed in alginate-treated discs. 

In our study, we ensured the application of clinically relevant products and models. We used beagles, a chondrodystrophic breed that closely resembles human disc organization and naturally experiences early-onset IVD degeneration and discogenic LBP—features not typically seen in other experimental animal models [67,75,77,78]. This similarity is speculated to be partly due to the loss of notochordal cells in the NP at stages of life similar to humans [79,80,81], unlike most other models (e.g., rats or rabbits) that retain these cells in large quantities into adulthood [81,82,83,84]. Notochordal cells are known for their higher regenerative capacity compared to NP cells, making our model ideal for assessing regenerative strategies due to the absence of these cells [81,82,83,84]. Our model consistently induced disc degeneration, though the severity was relatively mild and may not fully represent the typical clinical scenario. Additionally, our model involved acute degeneration, progressing over 2 weeks rather than the years or decades seen in human LBP patients [85,86,87]. Future work should aim to optimize this model by enhancing degeneration severity or allowing longer progression before treatment to better simulate chronic conditions. Finally, our study focused solely on degeneration without assessing pain or disability outcomes, which are the primary targets in clinical settings [77,88]. The lack of validated LBP measurements for experimental dogs and the administration of multiple treatments at different spinal levels limited such assessments in our design.

For our cell transplantation products, we employed an optimized culture method consistently shown to produce large quantities of highly potent cells. Following the work of Sako et al. [68], thecellular populations were found to have a high proportion of Tie2-positive cells, a specific NP progenitor cell population [71], with strong proliferative and regenerative characteristics [36,59,64,71,89,90]. Previous animal models have validated that such cell products can promote disc repair, in part by integrating long-term into treated discs [59,66]. However, further optimization may be considered by, for example, priming cells through growth factor stimulation to promote a higher ECM production potential [91,92,93]. Although, this would increase the complexity and price point of the cell therapy product, forming a hurdle for prospective marketability [5,94]. 

In our study, we also produced an OTS cell transplantation product and applied it directly from their cryopreserved state, following the optimal approach identified by Hiraishi et al. [66]. We stored our cells in CS-10 cryopreservation media, as this has been reported to maintain the highest cell viability and potency retention for Tie2-enhanced NP cell products [70,95]. Here, the CS-10 product contains 10% DMSO, which may present a cytotoxic impact on the transplanted and endemic disc cells [70,95,96,97]. Previous in vitro work [57], however, demonstrated that HA has the ability to limit DMSO-induced NP cell stress, which could help explain the beneficial outcomes for the HA cohort. Nevertheless, the impact of alginate on this DMSO-related cytotoxicity for the NP has not been studied. Finally, since our main aim was to compare the potential of biomaterials to enhance cell potency, we used a single donor for our cell transplantation product. It is important to note that using a different donor source could have influenced our results, as donor variability is a key factor in impacting regenerative outcomes [45,59,98,99].

### 4.1. Hyaluronic Acid Carrier

For the biomaterials, we used HA and alginate. Both materials are well-studied hydrogel products that have both been reported to enhance the potential of NP cells and promote their chondrogenic phenotype [61,63,64,93,100,101,102,103,104,105,106,107]. HA’s multifunctional properties, including its anti-inflammatory, anti-nociceptive, and ECM-modulating effects, position it as a promising candidate for IVD repair [52,103,108]. The evidence demonstrates that HA can effectively restore disc height, improve MRI signal intensity, and enhance ECM synthesis when used in both in vitro and in vivo models [103,108,109,110]. These findings underscore HA’s potential as a therapeutic agent in regenerative medicine for IVD repair. Aside from its application as a direct biomaterial agent, it is one of the most commonly applied carriers for intradiscal cellular therapeutics [5,111]. For example, the IDCT product by DiscGenics (USA), which encompasses allogenic colony-forming discogenic NP cells as their therapeutic agent, is infused in sodium hyaluronate solution, and found in their phase I/II clinical trial that their high cell density product could support significant improvements in LBP outcomes and support improvements in disc features [66,111,112]. Kumar, in their clinical trial, applied HA as a carrier for their adipose tissue-derived mesenchymal stromal cells (MSCs) and found evident improvement in pain and disability outcomes. Similarly, the Mesoblast Ltd. (Australia) MPC-06-ID product, involving mesenchymal precursor cells infused in 1% HA solution, has been shown in their phase II clinical trial to improve pain and disability outcomes, although no improvements were seen on MRI [113]. Notably, while statistically significant changes were observed, these results were also determined to be clinically relevant [5]. Interestingly, in both the Mesoblast Ltd. and DiscGenics trials, the HA-carrier-only control groups showed trends of improvement. However, the most significant changes were observed in treatments involving cells encapsulated within the hydrogel carriers. These findings align with our preclinical results, which demonstrated that while HA alone can limit disc degeneration, the most potent effects occurred when HA was loaded with our NP cell product. Furthermore, HA offers numerous opportunities for further refinement by adjusting the HA molecular weight used and its concentration, the potential of crosslinking, as well as through potential chemical modifications to enhance the biomimetic properties of the carrier and direct the desired cell behavior [58]. However, the full potential of such modifications requires further research. 

### 4.2. Alginate Carrier

For the usage of alginate as a carrier, no clinical trials have been reported that have employed intradiscal cell-based therapies [5]. Alginate, commonly derived from brown seaweed, is recognized for its biocompatibility and ability to mimic the ECM of the NP. Its ability to crosslink into three-dimensional networks provides mechanical support and maintains tissue hydration, which may be supportive of disc regeneration [60]. Studies have shown that alginate hydrogels can effectively support the delivery of cells, growth factors, and other therapeutic agents, leading to improved ECM synthesis and preservation of disc height in preclinical models [60]. Notably, alginate-based hydrogels have been utilized as carriers for MSCs and other progenitor cells. For example, in the work of Ura et al. [61], ultra-purified alginate gel implanted without cellular encapsulation could interfere with induced disc degeneration in their discectomy rat and rabbit models. Similarly, using their ultra-purified alginate gel loaded with MSCs could limit induced disc degeneration and discogenic pain in their rabbit models [100]. However, the interaction between alginate and NP cells appears to be complex and not always straightforward. Research has shown that while alginate provides a supportive environment for NP cells, it may also influence NP cell behavior in ways that could limit its therapeutic efficacy. For instance, Krouwels et al. [101,114] demonstrated that alginate hydrogels, depending on their stiffness and crosslinking, resulted in suboptimal NP cell proliferation and matrix synthesis, compared to a range of other hydrogel products. These findings suggest that the alginate’s physical properties may inadvertently alter NP cell activity, which may help explain why alginate did not function optimally in our study. Although alginate offers numerous opportunities for further refinement through chemical modifications, such as functionalizing the hydrogel with bioactive molecules to enhance cell adhesion and proliferation, the full potential of these modifications requires further research. 

The differences in outcomes between HA and alginate carriers observed in this study remain unspecified, but they likely stem from their distinct physicochemical and biological properties. HA, a key NP-ECM constituent, might better mimic the NP-matrix, supporting NP cell survival and metabolic activity through its hydrophilic nature and interactions with cell surface receptors such as CD44 [115]. These features promote cellular adhesion, proliferation, and ECM remodeling, all crucial for tissue repair. In contrast, we hypothesize that the alginate carrier, while providing mechanical stability, hydration, and uniform cell encapsulation, as a foreign entity in the NP environment may lack recognizable bioactive sites that limit endemic and encapsulated cell support for differentiation and tissue remodeling. Furthermore, the calcium ions required for crosslinking alginate may have suboptimal effects on the OTS-cell products. However, these considerations remain speculative. The superior regenerative outcomes with HA underscore the importance of selecting biomaterials that actively participate in biological processes and align with the specific needs of IVD regeneration [116]. Additionally, combined HA-alginate biomaterials have been constructed and might form a promising strategy to combine the best of both worlds in terms of their therapeutic potential [117].

Overall, our results demonstrate the potential of both biomaterial products and biomaterial-enhanced cell therapies to promote the restoration of IVD characteristics in a clinically relevant disc degeneration model. Notably, the combination of cells with HA showed the most promising results across all key outcome measures, though statistical significance compared to other treatment groups was often not achieved. Alginate alone led to significant deterioration in MRI hydration values, but this was mitigated by the addition of cell products. However, it is important to note that alginate with cells did not perform as well in other outcome assessments. This study offers valuable translational insights that can help guide the clinical development of cell-based therapies for improving outcomes in patients with LBP [5]. 

### 4.3. Limitations

While our study provides important insights into the use of alginate and HA as carriers for NP cell transplantation in a canine model, several limitations should be acknowledged. First, the use of a single donor source limits the generalizability of our findings, and the 12-week follow-up may be insufficient to capture long-term treatment effects [45,98,99,118]. Additionally, the acute degeneration model may not fully reflect the chronic nature of human disc degeneration, which could affect the relevance of our results [32,77,88]. The relatively mild degeneration induced by our aspiration method may have also tempered the observed therapeutic effects. Future studies should include more severely degenerated discs to better mimic clinical conditions and assess the full potential of the treatments to halt degeneration and promote regeneration [42]. Another limitation was the absence of baseline MRI measurements, which prevented us from fully evaluating the extent of induced degeneration. This decision was made to reduce harm and stress on the animals by minimizing the frequency of full sedation and handling, but it also meant we could not assess the initial health of the discs. Unexpectedly, we anticipated the healthy control discs to display consistent MRI results given the young age of the 1-year-old dogs, but even many unmanipulated discs showed signs of degeneration. This raises questions about whether this reflects early natural aging-related degeneration or whether there may be a breed-specific issue, or factors related to the breeding practices of our supplier [75,80,81]. Future studies should aim to assess disc hydration before inducing degeneration to fully understand the extent of degeneration that needs repair. Additionally, the 1.5T MRI used in this study lacked sufficient resolution to provide a clear view of the discs; we recommend using a 3T or higher MRI for more precise imaging in future work. Furthermore, our study did not assess pain or disability outcomes, which are critical in clinical contexts. Finally, our study only involved female canines. As sex-related differences in disc degeneration and back pain-related outcomes have been well-established, our results should be examined in male specimens for full validation [30,119,120]. Addressing these limitations will be key to advancing regenerative therapies for IVD degeneration.

## Data Availability

All data are presented in the manuscript or its supplementary files. Additional data can be requested from the corresponding authors upon reasonable request.

## Supplementary Materials

The following supporting information can be downloaded at https://www.mdpi.com/article/10.3390/cells13231984/s1, Supplementary Figure S1. Tracking of individual canine body weight throughout the study’s follow-up showed no evident negative changes over time; Supplementary Table S1. Tabular overview of average blood measurements pre- and post-transplantation; Supplementary Figure S2. Application of modified Pfirrmann classification scheme.

## Abbreviations

AF—Annulus fibrosis; CS-10—Cryostar-10; DHI—Disc height index; DMSO—Dimethyl sulfoxide; ECM—Extracellular matrix; FCM—Flow cytometry; HA—Hyaluronic acid; IVD—Intervertebral disc; LBP—Low back pain; MRI—Magnetic resonance imaging; MSC—Mesenchymal stromal cell; NP—Nucleus pulposus; ORS—Orthopaedic Research Society; OTS—Off-the-shelve; T2W—T2-weighted.

## References

[1] Young C.M., Quinn C., Trusheim M.R. (2022). Durable cell and gene therapy potential patient and financial impact: US projections of product approvals, patients treated, and product revenues. Drug Discov. Today.

[2] Takahashi T., Donahue R.P., Nordberg R.C., Hu J.C., Currall S.C., Athanasiou K.A. (2023). Commercialization of regenerative-medicine therapies. Nat. Rev. Bioeng..

[3] Yasuhara T., Kawauchi S., Kin K., Morimoto J., Kameda M., Sasaki T., Bonsack B., Kingsbury C., Tajiri N., Borlongan C.V. (2020). Cell therapy for central nervous system disorders: Current obstacles to progress. CNS Neurosci. Ther..

[4] Zelinka A., Roelofs A.J., Kandel R.A., De Bari C. (2022). Cellular therapy and tissue engineering for cartilage repair. Osteoarthr. Cartil..

[5] Schol J., Tamagawa S., Volleman T.N.E., Ishijima M., Sakai D. (2024). A comprehensive review of cell transplantation and platelet-rich plasma therapy for the treatment of disc degeneration-related back and neck pain: A systematic evidence-based analysis. JOR Spine.

[6] Rudnik-Jansen I., van Kruining Kodele S., Creemers L., Joosten B. (2024). Biomolecular therapies for chronic discogenic low back pain: A narrative review. JOR Spine.

[7] Härtl R., Bonassar L., Bonassar L.J. (2017). Biological Approaches to Spinal Disc Repair and Regeneration for Clinicians.

[8] Fournier D.E., Kiser P.K., Shoemaker J.K., Battie M.C., Seguin C.A. (2020). Vascularization of the human intervertebral disc: A scoping review. JOR Spine.

[9] McDonnell E.E., Buckley C.T. (2022). Two- and three-dimensional in vitro nucleus pulposus cultures: An in silico analysis of local nutrient microenvironments. JOR Spine.

[10] McDonnell E.E., Buckley C.T. (2022). Consolidating and re-evaluating the human disc nutrient microenvironment. JOR Spine.

[11] Crump K.B., Alminnawi A., Bermudez-Lekerika P., Compte R., Gualdi F., McSweeney T., Munoz-Moya E., Nuesch A., Geris L., Dudli S. (2023). Cartilaginous endplates: A comprehensive review on a neglected structure in intervertebral disc research. JOR Spine.

[12] Carreon L.Y., Ito T., Yamada M., Uchiyama S., Takahashi H.E. (1997). Neovascularization induced by anulus and its inhibition by cartilage endplate. Its role in disc absorption. Spine.

[13] McDonnell E.E., Wilson N., Barcellona M.N., Ni Neill T., Bagnall J., Brama P.A.J., Cunniffe G.M., Darwish S.L., Butler J.S., Buckley C.T. (2023). Preclinical to clinical translation for intervertebral disc repair: Effects of species-specific scale, metabolism, and matrix synthesis rates on cell-based regeneration. JOR Spine.

[14] Krouwels A., Popov-Celeketic J., Plomp S.G.M., Dhert W.J.A., Oner F.C., Bank R.A., Creemers L.B. (2018). No Effects of Hyperosmolar Culture Medium on Tissue Regeneration by Human Degenerated Nucleus Pulposus Cells Despite Upregulation Extracellular Matrix Genes. Spine.

[15] Fearing B.V., Hernandez P.A., Setton L.A., Chahine N.O. (2018). Mechanotransduction and cell biomechanics of the intervertebral disc. JOR Spine.

[16] Schol J., Sakai D., Warita T., Nukaga T., Sako K., Wangler S., Tamagawa S., Zeiter S., Alini M., Grad S. (2023). Homing of vertebral-delivered mesenchymal stromal cells for degenerative intervertebral discs repair—An in vivo proof-of-concept study. JOR Spine.

[17] Wang F., Cai F., Shi R., Wei J.N., Wu X.T. (2016). Hypoxia regulates sumoylation pathways in intervertebral disc cells: Implications for hypoxic adaptations. Osteoarthr. Cartil..

[18] Richardson S.M., Knowles R., Tyler J., Mobasheri A., Hoyland J.A. (2008). Expression of glucose transporters GLUT-1, GLUT-3, GLUT-9 and HIF-1alpha in normal and degenerate human intervertebral disc. Histochem. Cell Biol..

[19] Mern D.S., Beierfubeta A., Fontana J., Thome C., Hegewald A.A. (2014). Imbalanced protein expression patterns of anabolic, catabolic, anti-catabolic and inflammatory cytokines in degenerative cervical disc cells: New indications for gene therapeutic treatments of cervical disc diseases. PLoS ONE.

[20] Oichi T., Taniguchi Y., Oshima Y., Tanaka S., Saito T. (2020). Pathomechanism of intervertebral disc degeneration. JOR Spine.

[21] He S., Zhang Y., Zhou Z., Shao X., Chen K., Dai S., Liang T., Qian Z., Luo Z. (2022). Similarity and difference between aging and puncture-induced intervertebral disc degeneration. J. Orthop. Res..

[22] Kirnaz S., Capadona C., Wong T., Goldberg J.L., Medary B., Sommer F., McGrath L.B., Hartl R. (2022). Fundamentals of Intervertebral Disc Degeneration. World Neurosurg..

[23] Tamagawa S., Sakai D., Nojiri H., Nakamura Y., Warita T., Matsushita E., Schol J., Soma H., Ogasawara S., Munesada D. (2024). SOD2 orchestrates redox homeostasis in intervertebral discs: A novel insight into oxidative stress-mediated degeneration and therapeutic potential. Redox Biol..

[24] Li L., Al-Jallad H., Sun A., Georgiopoulos M., Bokhari R., Ouellet J., Jarzem P., Cherif H., Haglund L. (2024). The proteomic landscape of extracellular vesicles derived from human intervertebral disc cells. JOR Spine.

[25] Huang Y.C., Urban J.P., Luk K.D. (2014). Intervertebral disc regeneration: Do nutrients lead the way?. Nat. Rev. Rheumatol..

[26] Rustenburg C.M.E., Emanuel K.S., Peeters M., Lems W.F., Vergroesen P.A., Smit T.H. (2018). Osteoarthritis and intervertebral disc degeneration: Quite different, quite similar. JOR Spine.

[27] Diwan A.D., Melrose J. (2023). Intervertebral disc degeneration and how it leads to low back pain. JOR Spine.

[28] Zhang S., Hu B., Liu W., Wang P., Lv X., Chen S., Shao Z. (2021). The role of structure and function changes of sensory nervous system in intervertebral disc-related low back pain. Osteoarthr. Cartil..

[29] Lama P., Le Maitre C.L., Harding I.J., Dolan P., Adams M.A. (2018). Nerves and blood vessels in degenerated intervertebral discs are confined to physically disrupted tissue. J. Anat..

[30] Collaborators G.B.D.O.M.D. (2023). Global, regional, and national burden of other musculoskeletal disorders, 1990-2020, and projections to 2050: A systematic analysis of the Global Burden of Disease Study 2021. Lancet Rheumatol.

[31] Ambrosio L., Mazzuca G., Maguolo A., Russo F., Cannata F., Vadala G., Maffeis C., Papalia R., Denaro V. (2023). The burden of low back pain in children and adolescents with overweight and obesity: From pathophysiology to prevention and treatment strategies. Ther. Adv. Musculoskelet. Dis..

[32] Zhou M., Theologis A.A., O’Connell G.D. (2024). Understanding the etiopathogenesis of lumbar intervertebral disc herniation: From clinical evidence to basic scientific research. JOR Spine.

[33] Jiang W., Glaeser J.D., Kaneda G., Sheyn J., Wechsler J.T., Stephan S., Salehi K., Chan J.L., Tawackoli W., Avalos P. (2023). Intervertebral disc human nucleus pulposus cells associated with back pain trigger neurite outgrowth in vitro and pain behaviors in rats. Sci. Transl. Med..

[34] Harris I.A., Traeger A., Stanford R., Maher C.G., Buchbinder R. (2018). Lumbar spine fusion: What is the evidence?. Intern. Med. J..

[35] Schol J., Ambrosio L., Tamagawa S., Joyce K., Ruiz-Fernandez C., Nomura A., Sakai D. (2024). Enzymatic chemonucleolysis for lumbar disc herniation-an assessment of historical and contemporary efficacy and safety: A systematic review and meta-analysis. Sci. Rep..

[36] Ambrosio L., Schol J., Ruiz-Fernandez C., Tamagawa S., Soma H., Tilotta V., Di Giacomo G., Cicione C., Nakayama S., Kamiya K. (2024). ISSLS PRIZE in Basic Science 2024: Superiority of nucleus pulposus cell- versus mesenchymal stromal cell-derived extracellular vesicles in attenuating disc degeneration and alleviating pain. Eur. Spine J..

[37] Vadala G., Ambrosio L., Russo F., Papalia R., Denaro V. (2021). Stem Cells and Intervertebral Disc Regeneration Overview-What They Can and Can’t Do. Int. J. Spine Surg..

[38] Pers Y.M., Soler-Rich R., Vadala G., Ferreira R., Duflos C., Picot M.C., Herman F., Broussous S., Sanchez A., Noriega D. (2024). Allogenic bone marrow-derived mesenchymal stromal cell-based therapy for patients with chronic low back pain: A prospective, multicentre, randomised placebo controlled trial (RESPINE study). Ann. Rheum. Dis..

[39] Samanta A., Lufkin T., Kraus P. (2023). Intervertebral disc degeneration-Current therapeutic options and challenges. Front. Public Health.

[40] Zhang A., Cheng Z., Chen Y., Shi P., Gan W., Zhang Y. (2023). Emerging tissue engineering strategies for annulus fibrosus therapy. Acta Biomater..

[41] FDA Approved Cellular and Gene Therapy Products. https://www.fda.gov/vaccines-blood-biologics/cellular-gene-therapy-products/approved-cellular-and-gene-therapy-products.

[42] Loibl M., Wuertz-Kozak K., Vadala G., Lang S., Fairbank J., Urban J.P. (2019). Controversies in regenerative medicine: Should intervertebral disc degeneration be treated with mesenchymal stem cells?. JOR Spine.

[43] McDonnell J.M., Ahern D.P., Ross T.D., Morrissey P.B., Wagner S.C., Vaccaro A.R., Butler J.S. (2021). Regenerative Medicine Modalities for the Treatment of Degenerative Disk Disease. Clin. Spine Surg..

[44] Hwang P.Y., Chen J., Jing L., Hoffman B.D., Setton L.A. (2014). The role of extracellular matrix elasticity and composition in regulating the nucleus pulposus cell phenotype in the intervertebral disc: A narrative review. J. Biomech. Eng..

[45] McDonnell E.E., Ni Neill T., Wilson N., Darwish S.L., Butler J.S., Buckley C.T. (2024). In silico modeling the potential clinical effect of growth factor treatment on the metabolism of human nucleus pulposus cells. JOR Spine.

[46] Huang Y.C., Leung V.Y., Lu W.W., Luk K.D. (2013). The effects of microenvironment in mesenchymal stem cell-based regeneration of intervertebral disc. Spine J. Off. J. N. Am. Spine Soc..

[47] Yamada K., Iwasaki N., Sudo H. (2022). Biomaterials and Cell-Based Regenerative Therapies for Intervertebral Disc Degeneration with a Focus on Biological and Biomechanical Functional Repair: Targeting Treatments for Disc Herniation. Cells.

[48] Panebianco C.J., Meyers J.H., Gansau J., Hom W.W., Iatridis J.C. (2020). Balancing biological and biomechanical performance in intervertebral disc repair: A systematic review of injectable cell delivery biomaterials. Eur. Cells Mater..

[49] Croft A.S., Spessot E., Bhattacharjee P., Yang Y., Motta A., Woltje M., Gantenbein B. (2022). Biomedical applications of silk and its role for intervertebral disc repair. JOR Spine.

[50] Vadala G., Sowa G., Hubert M., Gilbertson L.G., Denaro V., Kang J.D. (2012). Mesenchymal stem cells injection in degenerated intervertebral disc: Cell leakage may induce osteophyte formation. J. Tissue Eng. Regen. Med..

[51] Zeng Y., Chen C., Liu W., Fu Q., Han Z., Li Y., Feng S., Li X., Qi C., Wu J. (2015). Injectable microcryogels reinforced alginate encapsulation of mesenchymal stromal cells for leak-proof delivery and alleviation of canine disc degeneration. Biomaterials.

[52] Kazezian Z., Joyce K., Pandit A. (2020). The Role of Hyaluronic Acid in Intervertebral Disc Regeneration. Appl. Sci..

[53] Vadala G., Russo F., Musumeci M., D’Este M., Cattani C., Catanzaro G., Tirindelli M.C., Lazzari L., Alini M., Giordano R. (2017). Clinically relevant hydrogel-based on hyaluronic acid and platelet rich plasma as a carrier for mesenchymal stem cells: Rheological and biological characterization. J. Orthop. Res..

[54] Zhang H., Zhang K., Zhang X., Zhu Z., Yan S., Sun T., Guo A., Jones J., Steen R.G., Shan B. (2015). Comparison of two hyaluronic acid formulations for safety and efficacy (CHASE) study in knee osteoarthritis: A multicenter, randomized, double-blind, 26-week non-inferiority trial comparing Durolane to Artz. Arthritis Res. Ther..

[55] Marinho A., Nunes C., Reis S. (2021). Hyaluronic Acid: A Key Ingredient in the Therapy of Inflammation. Biomolecules.

[56] Litwiniuk M., Krejner A., Speyrer M.S., Gauto A.R., Grzela T. (2016). Hyaluronic Acid in Inflammation and Tissue Regeneration. Wounds.

[57] Munesada D., Sakai D., Nakamura Y., Schol J., Matsushita E., Tamagawa S., Sako K., Ogasawara S., Sato M., Watanabe M. (2023). Investigation of the Mitigation of DMSO-Induced Cytotoxicity by Hyaluronic Acid following Cryopreservation of Human Nucleus Pulposus Cells. Int. J. Mol. Sci..

[58] Wang M., Deng Z., Guo Y., Xu P. (2022). Designing functional hyaluronic acid-based hydrogels for cartilage tissue engineering. Mater. Today Bio.

[59] Otani Y., Schol J., Sakai D., Nakamura Y., Sako K., Warita T., Tamagawa S., Ambrosio L., Munesada D., Ogasawara S. (2024). Assessment of Tie2-Rejuvenated Nucleus Pulposus Cell Transplants from Young and Old Patient Sources Demonstrates That Age Still Matters. Int. J. Mol. Sci..

[60] Jarrah R.M., Potes M.D.A., Vitija X., Durrani S., Ghaith A.K., Mualem W., Zamanian C., Bhandarkar A.R., Bydon M. (2023). Alginate hydrogels: A potential tissue engineering intervention for intervertebral disc degeneration. J. Clin. Neurosci. Off. J. Neurosurg. Soc. Australas..

[61] Ura K., Yamada K., Tsujimoto T., Ukeba D., Iwasaki N., Sudo H. (2021). Ultra-purified alginate gel implantation decreases inflammatory cytokine levels, prevents intervertebral disc degeneration, and reduces acute pain after discectomy. Sci. Rep..

[62] Lee K.Y., Mooney D.J. (2012). Alginate: Properties and biomedical applications. Prog. Polym. Sci..

[63] Christiani T., Mys K., Dyer K., Kadlowec J., Iftode C., Vernengo A.J. (2021). Using embedded alginate microparticles to tune the properties of in situ forming poly(N-isopropylacrylamide)-graft-chondroitin sulfate bioadhesive hydrogels for replacement and repair of the nucleus pulposus of the intervertebral disc. JOR Spine.

[64] Guerrero J., Hackel S., Croft A.S., Albers C.E., Gantenbein B. (2021). The effects of 3D culture on the expansion and maintenance of nucleus pulposus progenitor cell multipotency. JOR Spine.

[65] Bowles R.D., Setton L.A. (2017). Biomaterials for intervertebral disc regeneration and repair. Biomaterials.

[66] Hiraishi S., Schol J., Sakai D., Nukaga T., Erickson I., Silverman L., Foley K., Watanabe M. (2018). Discogenic cell transplantation directly from a cryopreserved state in an induced intervertebral disc degeneration canine model. JOR Spine.

[67] Thompson K., Moore S., Tang S., Wiet M., Purmessur D. (2018). The chondrodystrophic dog: A clinically relevant intermediate-sized animal model for the study of intervertebral disc-associated spinal pain. JOR Spine.

[68] Sako K., Sakai D., Nakamura Y., Schol J., Matsushita E., Warita T., Horikita N., Sato M., Watanabe M. (2021). Effect of Whole Tissue Culture and Basic Fibroblast Growth Factor on Maintenance of Tie2 Molecule Expression in Human Nucleus Pulposus Cells. Int. J. Mol. Sci..

[69] Basatvat S., Bach F.C., Barcellona M.N., Binch A.L., Buckley C.T., Bueno B., Chahine N.O., Chee A., Creemers L.B., Dudli S. (2023). Harmonization and standardization of nucleus pulposus cell extraction and culture methods. JOR Spine.

[70] Sako K., Sakai D., Nakamura Y., Matsushita E., Schol J., Warita T., Horikita N., Sato M., Watanabe M. (2021). Optimization of Spheroid Colony Culture and Cryopreservation of Nucleus Pulposus Cells for the Development of Intervertebral Disc Regenerative Therapeutics. Appl. Sci..

[71] Sakai D., Schol J., Bach F.C., Tekari A., Sagawa N., Nakamura Y., Chan S.C.W., Nakai T., Creemers L.B., Frauchiger D.A. (2018). Successful fishing for nucleus pulposus progenitor cells of the intervertebral disc across species. JOR Spine.

[72] Fujita N., Miyamoto T., Imai J., Hosogane N., Suzuki T., Yagi M., Morita K., Ninomiya K., Miyamoto K., Takaishi H. (2005). CD24 is expressed specifically in the nucleus pulposus of intervertebral discs. Biochem. Biophys. Res. Commun..

[73] Griffith J.F., Wang Y.X., Antonio G.E., Choi K.C., Yu A., Ahuja A.T., Leung P.C. (2007). Modified Pfirrmann grading system for lumbar intervertebral disc degeneration. Spine.

[74] Pfirrmann C.W., Metzdorf A., Zanetti M., Hodler J., Boos N. (2001). Magnetic resonance classification of lumbar intervertebral disc degeneration. Spine.

[75] Lee N.N., Salzer E., Bach F.C., Bonilla A.F., Cook J.L., Gazit Z., Grad S., Ito K., Smith L.J., Vernengo A. (2021). A comprehensive tool box for large animal studies of intervertebral disc degeneration. JOR Spine.

[76] Thompson J.P., Pearce R.H., Schechter M.T., Adams M.E., Tsang I.K., Bishop P.B. (1990). Preliminary evaluation of a scheme for grading the gross morphology of the human intervertebral disc. Spine.

[77] Alini M., Diwan A.D., Erwin W.M., Little C.B., Melrose J. (2023). An update on animal models of intervertebral disc degeneration and low back pain: Exploring the potential of artificial intelligence to improve research analysis and development of prospective therapeutics. JOR Spine.

[78] Bergknut N., Rutges J.P., Kranenburg H.J., Smolders L.A., Hagman R., Smidt H.J., Lagerstedt A.S., Penning L.C., Voorhout G., Hazewinkel H.A. (2012). The dog as an animal model for intervertebral disc degeneration?. Spine.

[79] Ambrosio L., Schol J., Ruiz-Fernandez C., Tamagawa S., Joyce K., Nomura A., de Rinaldis E., Sakai D., Papalia R., Vadala G. (2024). Getting to the Core: Exploring the Embryonic Development from Notochord to Nucleus Pulposus. J. Dev. Biol..

[80] Bach F.C., Poramba-Liyanage D.W., Riemers F.M., Guicheux J., Camus A., Iatridis J.C., Chan D., Ito K., Le Maitre C.L., Tryfonidou M.A. (2021). Notochordal Cell-Based Treatment Strategies and Their Potential in Intervertebral Disc Regeneration. Front. Cell Dev. Biol..

[81] Williams R.J., Laagland L.T., Bach F.C., Ward L., Chan W., Tam V., Medzikovic A., Basatvat S., Paillat L., Vedrenne N. (2023). Recommendations for intervertebral disc notochordal cell investigation: From isolation to characterization. JOR Spine.

[82] Bach F.C., Tellegen A.R., Beukers M., Miranda-Bedate A., Teunissen M., de Jong W.A.M., de Vries S.A.H., Creemers L.B., Benz K., Meij B.P. (2018). Biologic canine and human intervertebral disc repair by notochordal cell-derived matrix: From bench towards bedside. Oncotarget.

[83] Bach F.C., de Vries S.A., Krouwels A., Creemers L.B., Ito K., Meij B.P., Tryfonidou M.A. (2015). The species-specific regenerative effects of notochordal cell-conditioned medium on chondrocyte-like cells derived from degenerated human intervertebral discs. Eur. Cells Mater..

[84] Williams R.J., Tryfonidou M.A., Snuggs J.W., Le Maitre C.L. (2021). Cell sources proposed for nucleus pulposus regeneration. JOR Spine.

[85] Ruiz-Fernandez C., Francisco V., Pino J., Mera A., Gonzalez-Gay M.A., Gomez R., Lago F., Gualillo O. (2019). Molecular Relationships among Obesity, Inflammation and Intervertebral Disc Degeneration: Are Adipokines the Common Link?. Int. J. Mol. Sci..

[86] Johnson Z.I., Schoepflin Z.R., Choi H., Shapiro I.M., Risbud M.V. (2015). Disc in flames: Roles of TNF-alpha and IL-1beta in intervertebral disc degeneration. Eur. Cells Mater..

[87] Malik K.M., Cohen S.P., Walega D.R., Benzon H.T. (2013). Diagnostic criteria and treatment of discogenic pain: A systematic review of recent clinical literature. Spine J. Off. J. N. Am. Spine Soc..

[88] Poletto D.L., Crowley J.D., Tanglay O., Walsh W.R., Pelletier M.H. (2023). Preclinical in vivo animal models of intervertebral disc degeneration. Part 1: A systematic review. JOR Spine.

[89] Xia K.S., Li D.D., Wang C.G., Ying L.W., Wang J.K., Yang B., Shu J.W., Huang X.P., Zhang Y.A., Yu C. (2023). An esterase-responsive ibuprofen nano-micelle pre-modified embryo derived nucleus pulposus progenitor cells promote the regeneration of intervertebral disc degeneration. Bioact. Mater..

[90] Zhang X., Guerrero J., Croft A.S., Albers C.E., Hackel S., Gantenbein B. (2020). Spheroid-Like Cultures for Expanding Angiopoietin Receptor-1 (aka. Tie2) Positive Cells from the Human Intervertebral Disc. Int. J. Mol. Sci..

[91] Morita K., Schol J., Volleman T.N.E., Sakai D., Sato M., Watanabe M. (2021). Screening for Growth-Factor Combinations Enabling Synergistic Differentiation of Human MSC to Nucleus Pulposus Cell-Like Cells. Appl. Sci..

[92] Volleman T.N.E., Schol J., Morita K., Sakai D., Watanabe M. (2020). Wnt3a and wnt5a as Potential Chondrogenic Stimulators for Nucleus Pulposus Cell Induction: A Comprehensive Review. Neurospine.

[93] Fontana G., Thomas D., Collin E., Pandit A. (2014). Microgel microenvironment primes adipose-derived stem cells towards an NP cells-like phenotype. Adv. Healthc. Mater..

[94] Smith L.J., Iatridis J.C., Dahia C.L. (2020). Advancing basic and preclinical spine research: Highlights from the ORS PSRS 5th International Spine Research Symposium. JOR Spine.

[95] Croft A.S., Guerrero J., Oswald K.A.C., Hackel S., Albers C.E., Gantenbein B. (2021). Effect of different cryopreservation media on human nucleus pulposus cells’ viability and trilineage potential. JOR Spine.

[96] Chen X., Foote A.G., Thibeault S.L. (2017). Cell density, dimethylsulfoxide concentration and needle gauge affect hydrogel-induced bone marrow mesenchymal stromal cell viability. Cytotherapy.

[97] Shalash W., Forcier R., Higgins A.Z., Giers M.B. (2024). Cryopreserving the intact intervertebral disc without compromising viability. JOR Spine.

[98] Silverman L.I., Dulatova G., Tandeski T., Erickson I.E., Lundell B., Toplon D., Wolff T., Howard A., Chintalacharuvu S., Foley K.T. (2020). In vitro and in vivo evaluation of discogenic cells, an investigational cell therapy for disc degeneration. Spine J. Off. J. N. Am. Spine Soc..

[99] Wu H., Shang Y., Yu J., Zeng X., Lin J., Tu M., Cheang L.H., Zhang J. (2018). Regenerative potential of human nucleus pulposus resident stem/progenitor cells declines with ageing and intervertebral disc degeneration. Int. J. Mol. Med..

[100] Ukeba D., Sudo H., Tsujimoto T., Ura K., Yamada K., Iwasaki N. (2020). Bone marrow mesenchymal stem cells combined with ultra-purified alginate gel as a regenerative therapeutic strategy after discectomy for degenerated intervertebral discs. eBioMedicine.

[101] Krouwels A., Melchels F.P.W., van Rijen M.H.P., Ten Brink C.B.M., Dhert W.J.A., Cumhur Oner F., Tryfonidou M.A., Creemers L.B. (2018). Focal adhesion signaling affects regeneration by human nucleus pulposus cells in collagen- but not carbohydrate-based hydrogels. Acta Biomater..

[102] Arkesteijn I.T., Smolders L.A., Spillekom S., Riemers F.M., Potier E., Meij B.P., Ito K., Tryfonidou M.A. (2015). Effect of coculturing canine notochordal, nucleus pulposus and mesenchymal stromal cells for intervertebral disc regeneration. Arthritis Res. Ther..

[103] Isa I.L., Srivastava A., Tiernan D., Owens P., Rooney P., Dockery P., Pandit A. (2015). Hyaluronic Acid Based Hydrogels Attenuate Inflammatory Receptors and Neurotrophins in Interleukin-1beta Induced Inflammation Model of Nucleus Pulposus Cells. Biomacromolecules.

[104] Chen Y.C., Su W.Y., Yang S.H., Gefen A., Lin F.H. (2013). In situ forming hydrogels composed of oxidized high molecular weight hyaluronic acid and gelatin for nucleus pulposus regeneration. Acta Biomater..

[105] Peroglio M., Grad S., Mortisen D., Sprecher C.M., Illien-Junger S., Alini M., Eglin D. (2012). Injectable thermoreversible hyaluronan-based hydrogels for nucleus pulposus cell encapsulation. Eur. Spine J..

[106] Huang B., Zhuang Y., Li C.Q., Liu L.T., Zhou Y. (2011). Regeneration of the intervertebral disc with nucleus pulposus cell-seeded collagen II/hyaluronan/chondroitin-6-sulfate tri-copolymer constructs in a rabbit disc degeneration model. Spine.

[107] Park S.H., Cho H., Gil E.S., Mandal B.B., Min B.H., Kaplan D.L. (2011). Silk-fibrin/hyaluronic acid composite gels for nucleus pulposus tissue regeneration. Tissue Eng. Part. A.

[108] Mohd Isa I.L., Abbah S.A., Kilcoyne M., Sakai D., Dockery P., Finn D.P., Pandit A. (2018). Implantation of hyaluronic acid hydrogel prevents the pain phenotype in a rat model of intervertebral disc injury. Sci. Adv..

[109] Inoue M., Isa I.L.M., Orita S., Suzuki-Narita M., Inage K., Shiga Y., Norimoto M., Umimura T., Sakai T., Eguchi Y. (2021). An Injectable Hyaluronic Acid Hydrogel Promotes Intervertebral Disc Repair in a Rabbit Model. Spine.

[110] Yamamoto T., Suzuki S., Fujii T., Mima Y., Watanabe K., Matsumoto M., Nakamura M., Fujita N. (2021). Efficacy of hyaluronic acid on intervertebral disc inflammation: An in vitro study using notochordal cell lines and human disc cells. J. Orthop. Res..

[111] Gornet M.F., Beall D.P., Davis T.T., Coric D., LaBagnara M., Krull A., DePalma M.J., Hsieh P.C., Mallempati S., Schranck F.W. (2024). Allogeneic Disc Progenitor Cells Safely Increase Disc Volume and Improve Pain, Disability, and Quality of Life in Patients with Lumbar Disc Degeneration-Results of an FDA-Approved Biologic Therapy Randomized Clinical Trial. Int. J. Spine Surg..

[112] (2017). US National Library of Medicine—ClinicalTrials.gov. https://clinicaltrials.gov/ct2/show/NCT03347708.

[113] Amirdelfan K., Bae H., McJunkin T., DePalma M., Kim K., Beckworth W.J., Ghiselli G., Bainbridge J.S., Dryer R., Deer T.R. (2021). Allogeneic mesenchymal precursor cells treatment for chronic low back pain associated with degenerative disc disease: A prospective randomized, placebo-controlled 36-month study of safety and efficacy. Spine J. Off. J. N. Am. Spine Soc..

[114] Krouwels A., Melchels F.P.W., van Rijen M.H.P., Oner F.C., Dhert W.J.A., Tryfonidou M.A., Creemers L.B. (2018). Comparing Hydrogels for Human Nucleus Pulposus Regeneration: Role of Osmolarity During Expansion. Tissue Eng. Part. C Methods.

[115] Cheung B.C.H., Chen X., Davis H.J., Nordmann C.S., Toth J., Hodgson L., Segall J.E., Shenoy V.B., Wu M. (2024). Identification of CD44 as a key engager to hyaluronic acid-rich extracellular matrices for cell traction force generation and tumor invasion in 3D. Matrix Biol. J. Int. Soc. Matrix Biol..

[116] Zhang S., Dong J., Pan R., Xu Z., Li M., Zang R. (2023). Structures, Properties, and Bioengineering Applications of Alginates and Hyaluronic Acid. Polymers.

[117] Pangjantuk A., Kaokaen P., Kunhorm P., Chaicharoenaudomrung N., Noisa P. (2024). 3D culture of alginate-hyaluronic acid hydrogel supports the stemness of human mesenchymal stem cells. Sci. Rep..

[118] Silverman L.I., Flanagan F., Rodriguez-Granrose D., Simpson K., Saxon L.H., Foley K.T. (2019). Identifying and Managing Sources of Variability in Cell Therapy Manufacturing and Clinical Trials. Regen. Eng. Transl. Med..

[119] Kenawy H.M., Nunez M.I., Morales X., Lisiewski L.E., Burt K.G., Kim M.K.M., Campos L., Kiridly N., Hung C.T., Chahine N.O. (2023). Sex differences in the biomechanical and biochemical responses of caudal rat intervertebral discs to injury. JOR Spine.

[120] Mosley G.E., Wang M., Nasser P., Lai A., Charen D.A., Zhang B., Iatridis J.C. (2020). Males and females exhibit distinct relationships between intervertebral disc degeneration and pain in a rat model. Sci. Rep..

