# Supplementary material for: Alginate vs. Hyaluronic Acid as Carriers for Nucleus Pulposus Cells: A Study on Regenerative Outcomes in Disc Degeneration
Source: Cells. 2024 Nov 30;13(23):1984. doi: 10.3390/cells13231984 (PMC11639827; doi:10.3390/cells13231984)
Supplement: Supplementary file 1 [file cells-13-01984-s001.zip › cells-3336220-supplementary.pdf]

**Supplementary data** to "Alginate vs. Hyaluronic Acid as Carriers for Nucleus Pulposus Cells: A Study on Regenerative Outcomes in Disc Degeneration" by S. Ogasawara, J. Schol, et al.

**Supplementary Figure S1. Tracking of individual canine body weight throughout the study follow-up showed no evident negative changes overtime.** Body weight tracking revealed no evident changes suggestive of complications resulting from disc degeneration or cell/biomaterial injection procedures.

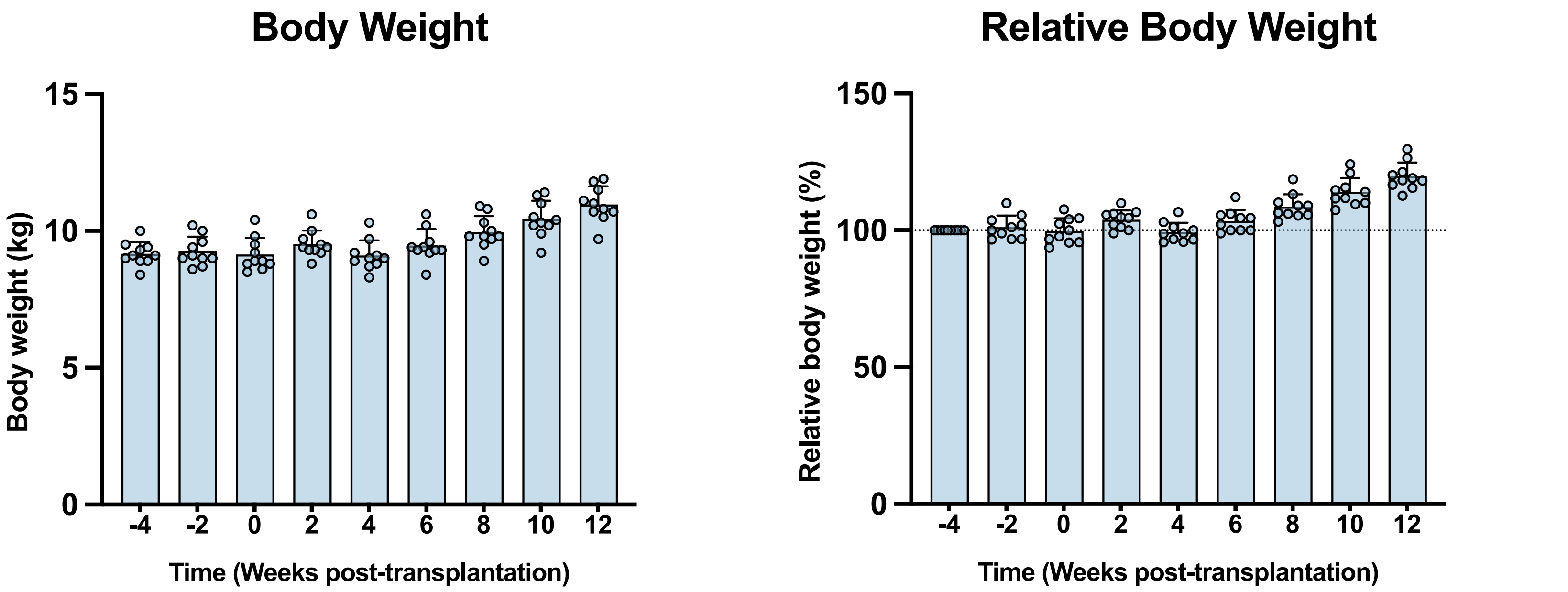

**Supplementary data** to "Alginate vs. Hyaluronic Acid as Carriers for Nucleus Pulposus Cells: A Study on Regenerative Outcomes in Disc Degeneration" by S. Ogasawara, J. Schol, et al.

**Supplementary Table S1. Tabular overview of average blood measurements pre- and post-transplantation**. Blood was drawn before and 4 weeks after the intradiscal injections. The results did not indicate any concerns, suggesting that the transplantation was well tolerated.

| | | Pre-transplantation | | | Post-transplantation | | | Average change | | |
|---|---|---|---|---|---|---|---|---|---|---|
| Type | (unit) | Average | Sd | N | Average | Sd | N | Average | Sd | N |
| Total Protein | (mg/dL) | 6.2 | 0.3 | 10 | 5.9 | 0.2 | 10 | -0.3 | 0.3 | 10 |
| Albumin | (mg/dL) | 2.9 | 0.1 | 10 | 2.8 | 0.1 | 10 | -0.2 | 0.1 | 10 |
| Urea Nitrogen | (mg/dL) | 14.8 | 2.4 | 10 | 15.4 | 2.5 | 10 | 0.6 | 3.2 | 10 |
| Creatinine | (mg/dL) | 0.6 | 0.1 | 10 | 0.7 | 0.1 | 10 | 0.1 | 0.1 | 10 |
| Cholestorol | (mg/dL) | 159 | 37.0 | 10 | 157 | 30.2 | 10 | -1.4 | 40.5 | 10 |
| Neutral fat | (mg/dL) | 22.0 | 8.1 | 10 | 19.0 | 8.5 | 10 | -3.6 | 10.6 | 10 |
| Bilirubin | (mg/dL) | 0.1 | 0.0 | 10 | 0.1 | 0.0 | 10 | 0.0 | 0.0 | 10 |
| AST | U/L | 30 | 5.6 | 10 | 42 | 11.0 | 10 | 11.7 | 13.0 | 10 |
| ALT | U/L | 36 | 11.2 | 10 | 35 | 9.9 | 10 | -1.1 | 8.3 | 10 |
| ALP | U/L | 283 | 77.1 | 10 | 81 | 29.0 | 10 | -202.5 | 56.9 | 10 |
| γ -GT | U/L | 4 | 1.0 | 10 | 4 | 0.8 | 10 | 0.0 | 1.1 | 10 |
| Sodium | mEq/L | 147 | 1.3 | 10 | 148 | 1.1 | 10 | 0.3 | 1.1 | 10 |
| Potassium | mEq/L | 5.0 | 0.2 | 10 | 4.8 | 0.3 | 10 | -0.2 | 0.3 | 10 |
| Chloride | mEq/L | 111 | 0.9 | 10 | 113 | 1.6 | 10 | 1.8 | 1.7 | 10 |
| Calcium | (mg/dL) | 9.3 | 0.6 | 10 | 10.0 | 0.5 | 10 | 0.7 | 0.4 | 10 |
| Inorganic phosphorus | (mg/dL) | 4.7 | 0.5 | 10 | 5.1 | 0.6 | 10 | 0.3 | 0.6 | 10 |
| Blood sugar | (mg/dL) | 92.4 | 8.1 | 10 | 81.8 | 5.1 | 10 | -10.6 | 6.8 | 10 |
| White blood cell count | WBC $x10^3/\mu L$ | 11.4 | 1.7 | 10 | 11.1 | 1.8 | 10 | -0.4 | 1.3 | 10 |
| Red blood cell count | RBC $x10^6/\mu L$ | 750.4 | 61.4 | 10 | 653.2 | 62.4 | 10 | -97.2 | 79.9 | 10 |
| Hemoglobin levels | g/dL | 17.3 | 1.4 | 10 | 14.8 | 1.6 | 10 | -2.5 | 2.0 | 10 |
| EVF | % | 52.5 | 3.5 | 10 | 45.9 | 3.1 | 10 | -6.7 | 4.7 | 10 |
| MCV | fL | 70.1 | 2.4 | 10 | 70.4 | 2.6 | 10 | 0.3 | 2.2 | 10 |
| MCH | pg | 23.0 | 0.6 | 10 | 22.7 | 0.4 | 10 | -0.4 | 0.6 | 10 |
| MHCH | g/dL | 32.9 | 0.9 | 10 | 32.2 | 1.5 | 10 | -0.7 | 1.3 | 10 |
| Platelet count | $x10^6/\mu L$ | 27.9 | 3.8 | 10 | 26.7 | 7.2 | 10 | -1.2 | 7.7 | 10 |
| PT | sec | 0.92 | 0.1 | 10 | 0.8 | 0.1 | 10 | -0.1 | 0.1 | 10 |
| PT activity | % | 9.6 | 1.3 | 10 | 8.9 | 0.7 | 10 | -0.7 | 1.1 | 10 |

Abbreviations: **ALP** - Alkaline Phosphatase; **ALT** - Alanine Aminotransferase; **AST** - Aspartate Aminotransferase; **EVF** - Erythrocyte Volume Fraction; **MCH** - Mean Corpuscular Hemoglobin; **MCV** - Mean Corpuscular Volume; **MCHC** - Mean Corpuscular Hemoglobin Concentration; **PT** - Prothrombin Time; **RBC** – Red blood cell; **sd** – standard deviation; **γ-GT** - Gamma-glutamyl Transferase; **WBC** – White blood cell.

**Supplementary data** to "Alginate vs. Hyaluronic Acid as Carriers for Nucleus Pulposus Cells: A Study on Regenerative Outcomes in Disc Degeneration" by S. Ogasawara, J. Schol, et al.

**Supplementary Figure S2. Application of modified Pfirrmann classification scheme.** (A) Modified Pfirrmann scores [1,2] applied to T2W sagittal MRI and (B) changes in Pfirrmann scores [1,2] between 0-week and 12-week's time points. Statistical analysis was performed using (A) 2-way ANOVA with Geisser-Greenhouse correction and (B, E) the Kruskal-Wallis test. For all conditions except the healthy controls (n=10), a sample size of six was involved. Bars represent mean values, dots indicate individual outcomes per disc, and error bars denote standard deviations.

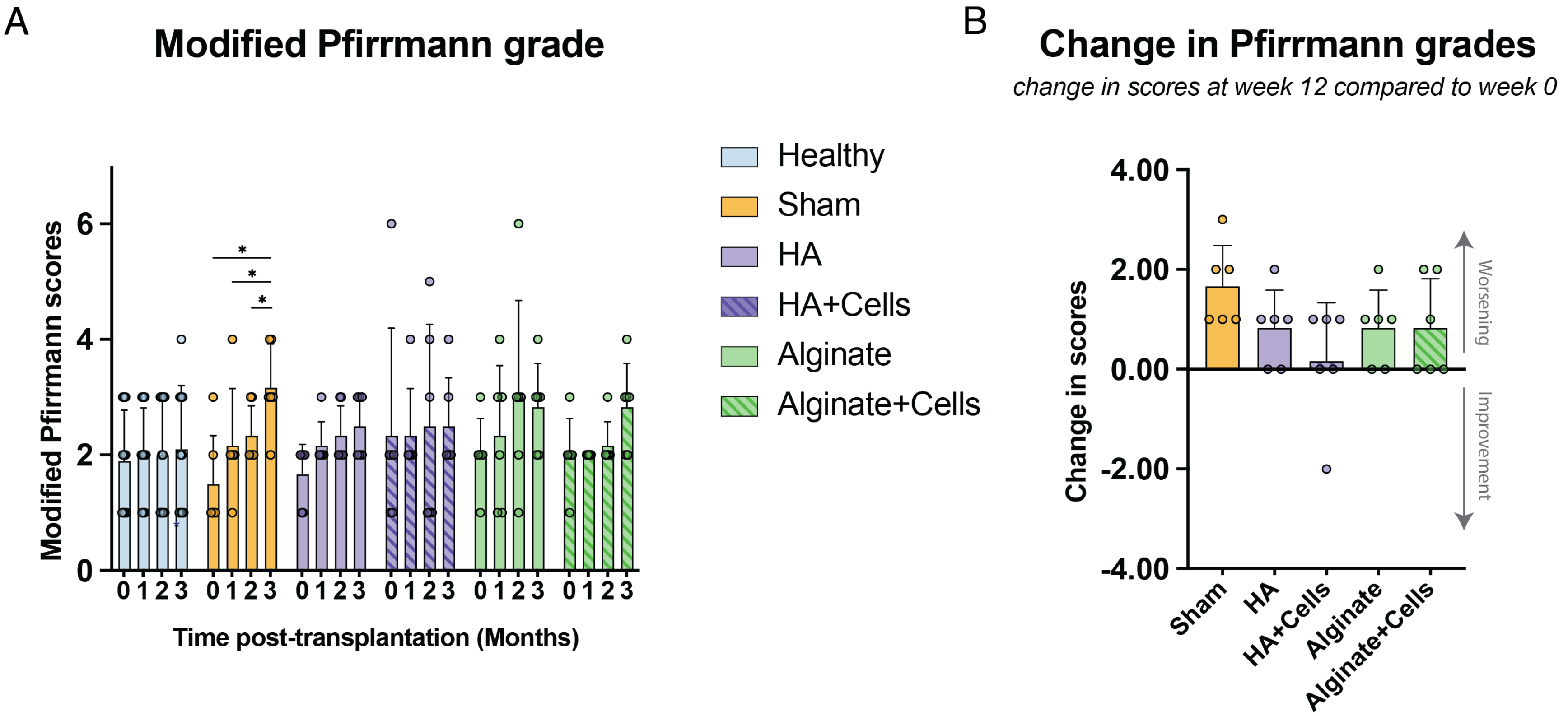

**Supplementary data** to "Alginate vs. Hyaluronic Acid as Carriers for Nucleus Pulposus Cells: A Study on Regenerative Outcomes in Disc Degeneration" by S. Ogasawara, J. Schol, et al.

REFERENCES


1. Griffith, J.F.; Wang, Y.X.; Antonio, G.E.; Choi, K.C.; Yu, A.; Ahuja, A.T.; Leung, P.C. Modified Pfirrmann grading system for lumbar intervertebral disc degeneration. *Spine (Phila Pa 1976)* **2007**, *32*, E708-712, doi:10.1097/BRS.0b013e31815a59a0.
2. Pfirrmann, C.W.; Metzdorf, A.; Zanetti, M.; Hodler, J.; Boos, N. Magnetic resonance classification of lumbar intervertebral disc degeneration. *Spine (Phila Pa 1976)* **2001**, *26*, 1873-1878, doi:10.1097/00007632-200109010-00011.
